# Performance Evaluation of Sandwich Structures Printed by Vat Photopolymerization

**DOI:** 10.3390/polym14081513

**Published:** 2022-04-08

**Authors:** Shukantu Dev Nath, Sabrina Nilufar

**Affiliations:** School of Mechanical, Aerospace, and Materials Engineering, Southern Illinois University Carbondale, Carbondale, IL 62901, USA; shukantudev.nath@siu.edu

**Keywords:** vat photopolymerization, sandwich panels, cellular core structures, dynamic mechanical analysis

## Abstract

Additive manufacturing such as vat photopolymerization allows to fabricate intricate geometric structures than conventional manufacturing techniques. However, the manufacturing of lightweight sandwich structures with integrated core and facesheet is rarely fabricated using this process. In this study, photoactivatable liquid resin was used to fabricate sandwich structures with various intricate core topologies including the honeycomb, re-entrant honeycomb, diamond, and square by a vat photopolymerization technique. Uniaxial compression tests were performed to investigate the compressive modulus and strength of these lightweight structures. Sandwich cores with the diamond structure exhibited superior compressive and weight-saving properties whereas the re-entrant structures showed high energy absorption capacity. The fractured regions of the cellular cores were visualized by scanning electron microscopy. Elastoplastic finite element analyses showed the stress distribution of the sandwich structures under compressive loading, which are found to be in good agreement with the experimental results. Dynamic mechanical analysis was performed to compare the behavior of these structures under varying temperatures. All the sandwich structures exhibited more stable thermomechanical properties than the solid materials at elevated temperatures. The findings of this study offer insights into the superior structural and thermal properties of sandwich structures printed by a vat photopolymerization technique, which can benefit a wide range of engineering applications.

## 1. Introduction

Sandwich panels are generally constructed with two facesheets separated by low-density core cellular structures. The facesheets and the core can be made of different or the same material. The mechanical properties of a sandwich panel depend mostly on the topology of the core cellular structures as well as the materials of the core and facesheets, and the geometry of the panel. The material of the facesheets and the topology of the cellular structure can be chosen according to the application of the panel, such as structural rigidity [1], energy absorption capacity [2], vibration and acoustic attenuation [3], thermal insulation property [4], etc. Sandwich panels are extensively utilized in airplane wings and bodies, lightweight sportswear, marine and military applications, thermal insulative walls/roofs, vibration absorbing materials, and automotive parts [5,6,7,8,9,10].

Foams are first studied as the core materials among all other core types of sandwich panels [11]. Foams are an excellent choice for compressive loading [12,13,14] but they offer poor bending performance due to their bending-dominated architecture [15], and exhibit prominent size effects [16]. Later, the randomly porous foam cores are replaced by defined periodic cellular architectures. Among those, the most studied shape is the conventional honeycomb structure. Honeycomb core is lightweight, strong, and a high energy absorbent in the out-of-plane direction [17,18,19,20]. Rectangular and diamond shapes are also commonly studied structures because of their simplicity and highly attractive physical properties. Studies suggested that a sandwich panel with the diamond structure is superior to that of the honeycomb structure in flexural tests in both in-plane and out-of-plane directions [21]. Recently, auxetic structures have been focused on as cellular cores of sandwich panels because of their unusual physical properties. Auxetic structures have a negative Poisson’s ratio. When stretched or compressed, they accordingly become thicker or thinner perpendicular to the direction of the applied force [22]. Theoretically, the auxetic structures do not offer high stiffness and strength and may not be as lightweight as other conventional structures. Nevertheless, these structures offer some engineering advantages, such as high energy absorption capacity [23], high deformation fracture toughness [24], shear resistance [25], and excellent impact and ballistic resistance [26,27]. Recent studies demonstrated that auxetic structures can be utilized to prepare excellent phononic crystals and acoustic metamaterials [28,29]. The re-entrant honeycomb structure has been investigated commonly among different kinds of auxetic structures for a range of deformation [26,30,31].

The conventional manufacturing of sandwich panels involves multiple stages of fabrication, offers very limited types of sandwich core topologies, and less design flexibility. The use of additive manufacturing techniques to fabricate the cores and the sandwich panels can eradicate some of these problems. The additive manufacturing techniques are classified into seven categories, such as binder jetting, directed energy deposition, material extrusion, material jetting, powder bed fusion, sheet lamination, and vat photopolymerization [32]. Additive manufacturing techniques offer an easy solution for manufacturing complex cellular cores. However, two identical samples of the same material, one fabricated conventionally and the other fabricated by additively manufacturing, can have a significant difference in mechanical properties [33,34]. Moreover, the same materials printed with different additive manufacturing techniques can differ in properties [35,36]. Several factors, such as different printing orientations and settings, can affect the properties of the final printed part [37,38,39,40,41]. Therefore, optimization of operating parameters and characterization of the printed parts for a specific structure is very crucial.

Several studies were conducted to evaluate the properties of sandwich panels printed by different 3D printing techniques. A study of 3D-printed sandwich panels with various cellular structures printed by a material extrusion process reported the re-entrant honeycomb structure to have more than two folds higher compressive strength than the conventional honeycomb structure [42]. The re-entrant honeycomb structure showed a periodic drop of stress after the failure of each layer during the compression test. Due to this type of failure, this structure tends to have a higher energy absorption capacity compared to regular shapes [43]. Li and Wang applied the material jetting additive manufacturing technique to print sandwich panels with honeycomb, re-entrant honeycomb, and diamond cellular structures [44]. The study reported the highest compressive modulus and strength for the honeycomb structure, whereas the re-entrant honeycomb structure showed an auxetic behavior. These different structures showed a very different mode of failure in flexural tests as well. The honeycomb and the diamond structures failed locally under flexural loading whereas the re-entrant honeycomb structure exhibited global failure. Zaharia and coworkers tested sandwich panels with honeycomb, diamond, and corrugated core structures printed by the material extrusion process [45]. The panels with diamond-celled structures showed the highest compressive and flexural strength whereas the corrugated structure showed the highest tensile strength. Due to the difference in the printing process and design considerations, printed samples exhibited different natures.

Studies of the properties of different sandwich panels have been conducted for different 3D printing techniques. However, there is still a lack of understanding of the mechanical behavior of sandwich panels with different cellular cores printed by the vat photopolymerization technique. The resolution, accuracy, and surface finish of the printed parts in vat photopolymerization are higher compared to other 3D printing techniques. However, the printing time is slow compared to other methods [41]. Therefore, the innovative nature of our work is encompassed around creating intricate sandwich structures using the vat photopolymerization process. In this study, we focused on characterizing the thermomechanical properties of these sandwich panels with conventional honeycomb, re-entrant honeycomb, diamond, and square cellular structures. Herein, uniaxial compression tests of different sandwich panels were conducted to find the compressive performance and specific strength of each type of panel. Finite element analyses were carried out to have a better understanding of the stress distribution due to compressive loading. Lastly, dynamic mechanical analysis (DMA) of the sandwich panels was conducted to investigate the change of properties under varying temperatures.

## 2. Materials and Methods

### 2.1. Structural Design

**Figure 1:**
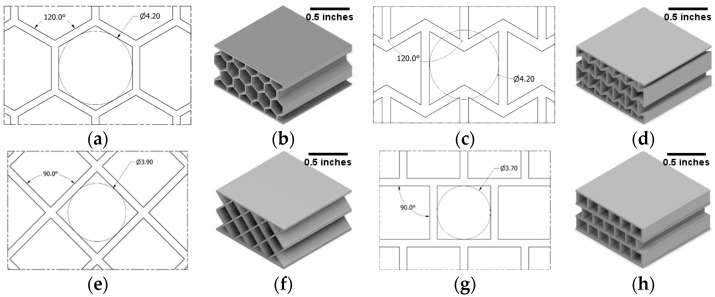
Design of unit cells of (**a**) honeycomb, (**c**) re-entrant honeycomb, (**e**) diamond, and (**g**) square. The wall thickness of all cellular walls and face walls is set to 0.5 mm. (**b**,**d**,**f**,**h**) are the complete CAD drawings of sandwich panels with different cellular structures.

The CAD models of the sandwich structures were prepared in Autodesk Inventor Professional 2020. The compression and tensile test samples were drawn according to ASTM D695-15 [46] and ASTM D638-14 [47]. For the sandwich panels, internal cellular structures of honeycomb, re-entrant honeycomb, square, and diamond were selected. Sandwich panels with these cellular structures were printed according to ASTM C365/C365M-16 [48]. All of the sandwich samples were designed to be exactly the same in their outer dimensions to avoid any geometric scaling effect on the properties [49]. Samples for DMA were prepared according to ASTM D7028-07 [50], with one layer of unit cells in between two facesheets. The facesheets and the internal walls of all the samples have a wall thickness of 0.5 mm. The cell sizes were optimized in both vertical and horizontal directions for each type of cell to avoid any dissimilarity in boundary cells. The design of the internal cellular structures and the CAD models of the sandwich panels are shown in Figure 1.

### 2.2. Fabrication of the Samples

**Figure 2:**
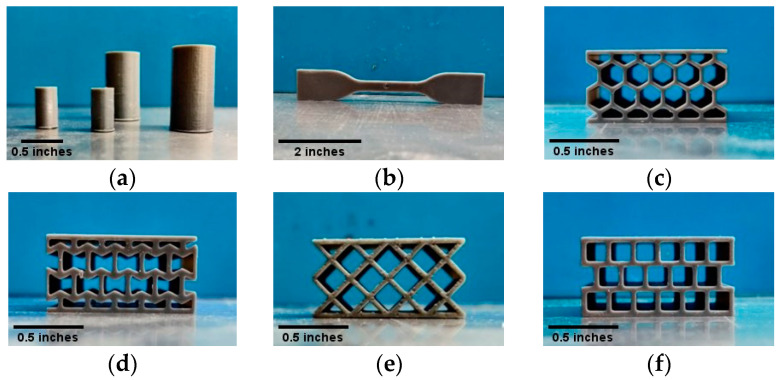
(**a**) Compression samples of different sizes according to ASTM D695-15 (**b**) Tensile sample according to ASTM D638-14. Sandwich structures with (**c**) honeycomb, (**d**) re-entrant honeycomb, (**e**) diamond, and (**f**) square cellular cores are printed according to ASTM C365/C365M-16. All the samples are printed with the same printing parameter and are from the same batch of resin.

The test specimens were fabricated using a desktop 3D printer, the MP Mini Deluxe 3D from Monoprice. The printer prints with a UV light having a 405 nm wavelength. A photocurable acrylic resin, ‘MP Rapid Gray’, rated for 405 nm, was used as the printing material. All the samples were printed with the same batch of resin to avoid any variation in the material. The CAD files of the samples were sliced in Creation Workshop slicing software. The layer thickness was set to 50 microns for all the samples. The printer follows a bottom-up printing process. After printing each layer, the printing base retracts to allow the new resin to enter between the newly printed layer and the floor of the resin tank. A suction force is generated during this time. Due to this force, the base layer may lose adhesion with the printing base, resulting in failed printing. Therefore, the base layer should adhere strongly to the printing base to hold the printing properly. A curing time of 60 s per layer for the base layers showed the least print failure and held the printed samples properly. For the rest of the printing, the curing time per layer was set to 10 s. The base layers were separated from the samples after printing as the samples were printed with removable supports. Table 1 shows the printing parameter. The direction of printing was kept the same for all the samples. Photocurable resins can be printed at room temperature and the printing process does not generate a significant amount of heat to generate residual stress. Therefore, all the samples were printed at room temperature. After printing, the samples were cleaned in isopropyl alcohol for 5 min. An optimized cleaning time improved the quality of the printed surface and the properties of the printed part [51]. The time duration of cleaning the printed resin in isopropyl alcohol should be optimized; otherwise, it can deteriorate the property of the samples [52]. Cleaned samples were post-treated into a UV light chamber for 1 h [53]. Figure 2 shows the printed compression, tensile, and sandwich structures including honeycomb, re-entrant honeycomb, diamond, and square specimens. If there are a significant number of gaps in the printed part, it may contribute to the overall structural properties. The printed samples considered for testing were selected carefully by optical microscopy to avoid samples with printing defects.

### 2.3. Mechancial and Thermomechancial Testing

A 30 kN MTS Universal testing machine was used for compression and tensile tests. Following the standard, the crosshead speed for the compression test was set to 1.3 mm/min for solid samples and 0.5 mm/min for the sandwich panels. The crosshead speed for the tensile tests was 5 mm/min. Tests were run until the samples reached fracture points. DMA was conducted with Discovery DMA 850 from TA Instruments. Three point-bending modes were utilized for the tests. The tests were performed in temperature ramp mode from 30 °C to 120 °C at a heating ramp of 5 °C/min to study the variation of the thermomechanical properties of the solid and sandwich samples. The amplitude was set to 30 µm and periodic loading was applied with a 1 Hz frequency. The storage modulus (*E΄*) and the mechanical damping factor (tan *δ*) were obtained from the DMA. The glass transition temperature (*T_g_*) was also calculated from the storage modulus vs. temperature graph. Scanning electron microscopy (SEM) images of the tested specimens facilitated the explanation of the failure under applied loading and stress concentration areas.

### 2.4. Finite Element Analysis

The numerical simulations related to the mechanical response of the sandwich panels under uniaxial compression loading were conducted using the commercially available ANSYS Workbench 2020 R2 (ANSYS Inc. Canonsburg, PA, USA). To simulate the material, the dimension of the specimens, material properties of the specimen, including the compressive and tensile properties of the solid samples, were introduced into the software. The Youngs modulus and yield strength were obtained from the uniaxial tensile test whereas the ultimate compressive strength was obtained from compression tests. The Poisson’s ratio was set to 0.33. To simulate the compression test, a fixed support boundary condition was set at one end and a gradual displacement conforming with the strain rate of the experimental study was set on the other end of the CAD modeled samples.

## 3. Results and Discussion

### 3.1. Mechancial Performance of Solid Materials

**Figure 3:**
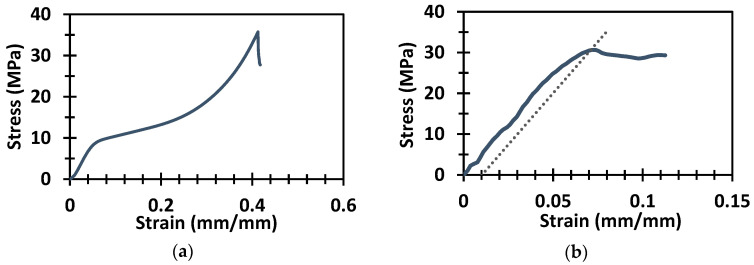
The stress–strain plots of the (**a**) compression test and (**b**) tensile test of the solid samples.

The compression tests of five solid samples printed by vat photopolymerization were conducted and the average compressive strength and modulus were determined from the test results. Figure 3a shows a representative stress–strain curve of the compression test. The compressive modulus and strength of the printed solid samples were around 0.2 GPa and around 34 MPa, respectively. The tensile tests were conducted to find the yield strength of the solid sample. Figure 3b shows a typical stress–strain graph of the tensile test of a solid sample. The yield strength calculated from the tensile test was 30.65 MPa. For polymers, the yield strength was found by the 1% offset method [54].

### 3.2. Effect of Core Topology on the Compressive Performance of Sandwich Structures

**Figure 4:**
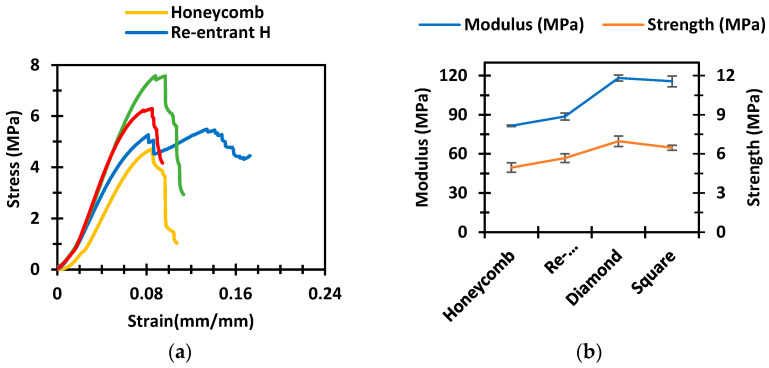
(**a**) The stress–strain plots of different sandwich panels. (**b**) The comparison of compressive strength and modulus of the sandwich panels.

The compressive properties of different sandwich panels vary with different cellular core structures. Figure 4a shows the representative stress–strain plots of the sandwich panels with honeycomb, re-entrant honeycomb, diamond, and square core topologies. The fracture stresses of all the structures fell within a small strain range, between 0.008 mm/mm to 0.012 mm/mm. The stresses of the honeycomb, diamond, and square structures dropped immediately after the first crack. Therefore, these structures show catastrophic damage at the sign of the first crack. In contrast, the re-entrant honeycomb structure did not fail catastrophically after the first crack as the structure held its integrity and took more load. The stress–strain curve continued to progress by holding the stress even after the first fracture point, which is typically not observed for any other cellular cores. Therefore, the re-entrant honeycomb structure can absorb more energy than any other structure due to the failure characteristics mentioned above. The energy absorption by honeycomb, re-entrant honeycomb, diamond, and square core topologies were found to be 380 kJ/m^3^, 1356 kJ/m^3^, 811 kJ/m^3^, and 611 kJ/m^3^, respectively. The energy absorption of the re-entrant honeycomb was 256% higher than that of the honeycomb structure.

Figure 4b represents the comparison of the compressive modulus and strength of different sandwich structures. The error bars represent the standard error of the mean. The honeycomb sandwich structure had the lowest compressive modulus and strength. The re-entrant honeycomb structure was slightly higher than the honeycomb structure in terms of both modulus and strength. The diamond structure had the highest modulus and strength. Compared to the honeycomb structures, the diamond structures showed around 45% higher modulus and 41% higher strength. The strength and modulus of the square structures were about the same compared to the diamond structures. Table 2 shows the average compressive modulus and strength with standard errors of five samples from each set of sandwich structures.

**Figure 5:**
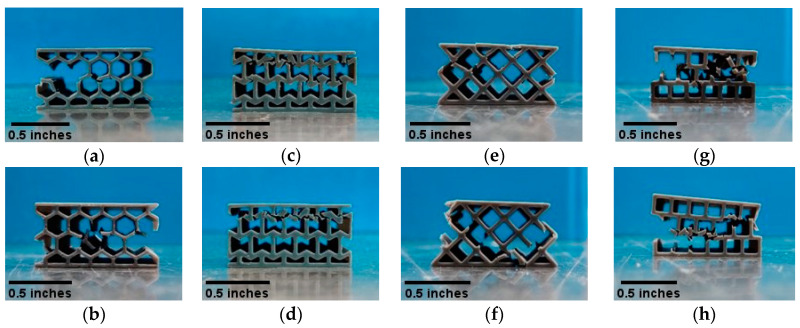
Representative images showing the failure after the compression test. (**a**,**b**) honeycomb; (**c**,**d**) re-entrant honeycomb; (**e**,**f**) diamond; (**g**,**h**) square sandwich panels.

Figure 5 shows the failed specimens of the sandwich panels after the compression test. The fractures of the honeycomb structure are at specific locations (Figure 5a,b). Damage within the cell walls of one or multiple adjacent cells caused the sudden failure of the honeycomb structures. Because of the localized failure of the honeycomb structures, they do not exhibit good compressive strengths compared to other structures.

The re-entrant honeycomb samples showed an interesting feature. Whereas other structures showed a sudden drop of stress after the fracture point, the re-entrant honeycomb structures maintained the stress. Under compressive loading, the re-entrant honeycomb structures showed a global failure mode (Figure 5c,d). Each layer of the cores failed one at a time under compression loading, leading up to a higher strain of failure of the samples. After the failure of each layer, the structures came to an equilibrium and could take further loading until the failure of the next layer. This phenomenon is often referred to as snap-through instability [55]. Snap-through instability is utilized for designing materials with multistable structures under compressive and tensile loading [56,57,58,59]. A study on the bending behavior of the re-entrant structure reported that this kind of failure provides a high energy absorption capacity of the sandwich structures over a large deformation [44]. The study also showed that the re-entrant cellular structure is auxetic in nature and exhibits multistable characteristics. The predictive nature of this layer-by-layer failure makes it appropriate for many engineering applications.

**Figure 6:**
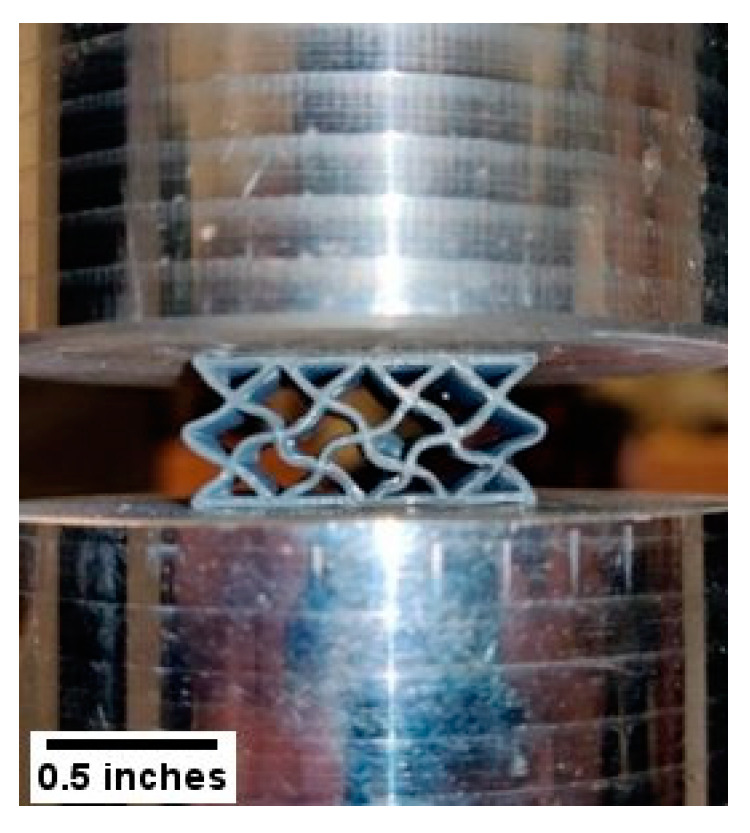
Bending of the cell walls of the diamond structure under compressive loading.

Figure 5e,f shows the fractures of the diamond structures observed at several places. The diamond structure distributed the compressive load more efficiently than any other structure. Figure 6 shows that the cell walls of the structure are slightly bent under compressive loading; this helps to gain a more delocalized distribution of the stress across the whole structure. This distribution of stress prevented the failure of any single specific section of the sample. The distribution of the compressive stress in diamond samples had effects on the facesheets as well. The facesheets of the diamond sandwich panels fractured with compressive loading, which is not observed for any other sandwich panels. This uniform distribution of stress can be attributed to the high compressive strength of this structure. Consistent with our findings, a recent study with the diamond cores reported that the diamond structures have greater compressive modulus and strength than the honeycomb and the corrugated structures [45]. The fracture of the square structures is also observed throughout the panel (Figure 5g,h).

### 3.3. Numerical Analysis

**Figure 7:**
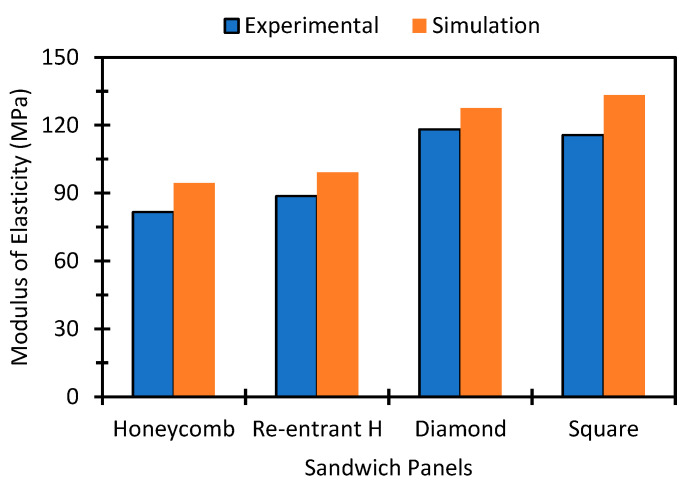
Comparison of the compressive modulus of the experimental and simulation results of different sandwich panels.

Figure 7 represents the comparison of the stress–strain results simulated by finite element analysis and experimental study. The simulated results show good agreement with the experimental results. However, the modulus found at the start of the compression test shows a lower value than the simulation value which may be due to the presence of layer gaps and microvoids. A homogenous mechanical property of any additively manufactured part is difficult to achieve due to the inclusion of imperfections such as microvoids, impurities, or defects during the printing process [60,61,62].

**Figure 8:**
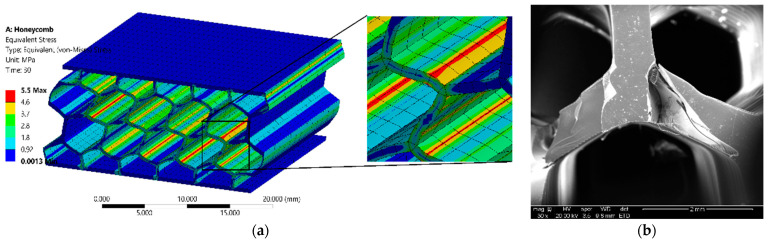

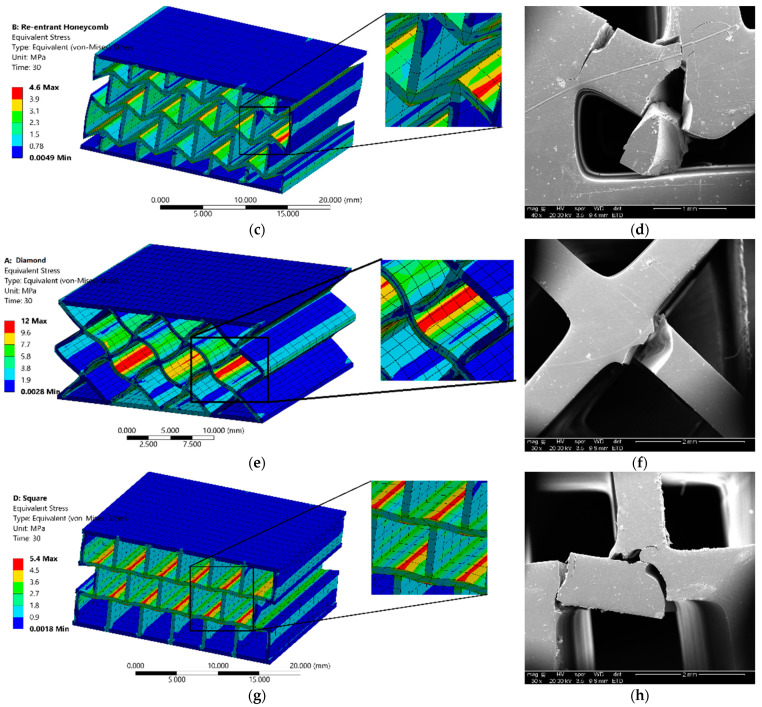
(**a**,**c**,**e**,**g**) Simulations up to the fracture strain of the modeled samples reveal the stress concentration locations of each type of sample. Stress concentration areas are further visualized at higher magnification. (**b**,**d**,**f**,**h**) SEM images of the tested samples show the most common locations of fracture of the samples.

The stress distributions of the sandwich structures generated by the simulation were utilized to identify the stress concentration areas in different sandwich panels under compressive loading along with corresponding SEM images of the fractured areas (Figure 8). The simulation of the sandwich panels in Figure 8a,c,e,g indicates that the stress concentration areas of different structures are adjacent to the cell wall joints. The SEM images of the samples in Figure 8b,d,f,h show the corresponding fractured areas across different structures after compression testing. The most frequently fractured areas are at the highest stress concentration locations indicated by the simulation study and are also evident from the SEM micrographs of fractured samples.

### 3.4. Effect of Relative Density

**Figure 9:**
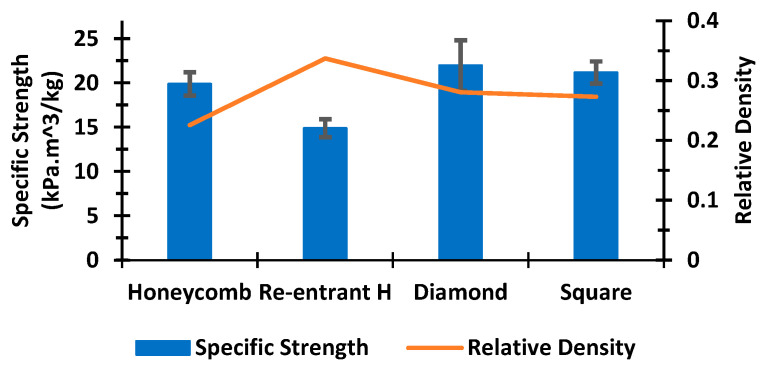
Relative comparison of specific strength and relative density of different sandwich structures.

The specific strength of each kind of sandwich panel was determined to compare the performance of different cores in weight-sensitive applications. For a specific core topology, the relative density depends on the slenderness ratio of the sides of the cores and the thickness of the facesheets. Figure 9 demonstrates a graphical comparison of the average specific strengths and relative densities of different sandwich panels. According to our design, the honeycomb structure is the lightest among all the sandwich panels, with a relative density of 0.23. The specific strength of the honeycomb structure was 19.88 kPa.m^3^/kg. The diamond cellular structure having a relative density of 0.28 showed an average specific strength of 21.96 kPa.m^3^/kg. The diamond and the square structure showed similar values in terms of both the relative density and the specific strength. The re-entrant structure was denser compared to the other structures, resulting in a lower specific strength. It is important to remember that the specific strength of the sandwich structure is highly dependent on design considerations, such as the cell size, wall thickness, number of cellular cores, and height-width ratio of the structure.

### 3.5. Dynamic Mechanical Analysis

**Figure 10:**
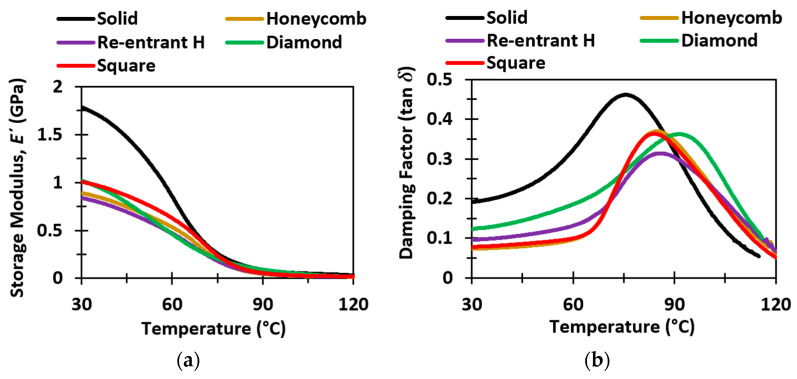
Comparison of the (**a**) storage modulus and (**b**) damping factor of the solid material and sandwich structures for a temperature ramp mode from 30 °C to 120 °C.

The DMA results of the sandwich structures revealed the change of dynamic mechanical properties due to the change of temperature. DMA is utilized to address the interfacial bonding and thermal relaxation of polymers and composites with stress, strain rate, and temperature. Even a small change in the physical nature of the materials results in a significant change in overall dynamic mechanical properties [63,64,65,66]. In our study, as the sandwich structures possessed different core topologies, the DMA results may reveal the effects of oscillating strain and varying temperature on the mechanical behavior. Figure 10a,b demonstrate the change of the storage modulus and the damping factor of different sandwich panels along with the solid samples, respectively. The storage modulus of the solid resin was higher than any of the sandwich panels at room temperature. However, with the temperature rise, the storage modulus began to decrease rapidly. At about 70 to 75 °C, the storage modulus of the solid sample matched with those of the sandwich panels. This quick drop of storage modulus of the solid sample resulted in a glass transition temperature (*T_g_*) of 53 °C. On the contrary, the honeycomb, re-entrant honeycomb, diamond, and square sandwich panels had superior glass transition temperatures (*T_g_*) of around 63.2 °C, 60.89 °C, 63.69 °C, and 63.80 °C, respectively. The storage modulus results of the sandwich panels exhibited a similarity with the increasing temperature and do not decrease as sharply as the solid sample.

The damping factor represented the ratio of energy dissipation per cycle of loading and maximum stored elastic energy within the material [67]. The lower value of the damping factor was associated with the improved load-bearing capacity of the material [63]. The damping factor of the solid material reached as high as 0.46 at around 75 °C, which is the highest of all the samples. The damping factor of any sandwich panel was significantly lower (ranges between 0.32–0.36) than the solid material at a lower temperature (<90 °C). Therefore, the softening of the solid material was more prominent than the sandwich structure. The low damping factor (<90 °C) of the sandwich panels indicated better mechanical performance with thermal stability. The re-entrant structure showed the lowest peak of the damping factor (~0.32). Therefore, it can be concluded that the re-entrant structure is more stable at elevated temperatures. It might be due to the easy removal of trapped heat through the core topology. The peak damping factor of the diamond structure had a similar value (~0.36) as the honeycomb and the square samples, but it was observed at a higher temperature, suggesting pronounced structural stability at an elevated temperature.

## 4. Conclusions

In this study, sandwich core structures with four different topologies with honeycomb, re-entrant honeycomb, diamond, and square were designed and fabricated by the vat photopolymerization technique. The structural properties of these structures under compressive loading were investigated through both experimental testing and numerical finite element analysis. The diamond structure showed the highest compressive modulus and strength. The honeycomb, diamond, and square structures showed catastrophic failure under compressive loading. The tested samples revealed that honeycomb and square structures failed with the collapse of cores in different specific locations. The diamond structure showed a bending tendency of the core walls under loading, which helped to distribute stress throughout the sample. Facesheets of diamond structures also fractured during compressive loading. The re-entrant honeycomb samples showed a failure throughout each layer of the core structure because of relatively homogenous stress distribution leading to a higher strain resistance and predictable fracture behavior. This behavior of the re-entrant honeycomb structure significantly increased the energy absorption capacity of the structure. The experimental and numerical results showed good agreement in terms of the stress–strain data, stress concentration, and deformation patterns. DMA results indicated that the sandwich panels have significantly lower storage modulus in comparison to the solid structures, but it is more stable at higher temperatures. The structural properties of these sandwich panels can be tailored by changing various factors, including core topology, wall thickness, and facesheets. These properties can also vary due to changes in the printing technique of the structures. The findings of our work provide insights into the development of the sandwich panels printed by vat photopolymerization technique for a wide range of engineering applications where lightweight materials with tailored structural properties are required.

## Figures and Tables

**Table 1:** Printing parameter of the samples.

Parameter	Value
Slice thickness	50 µm
Curing time of the base layer	60 s
Number of base layers	4
Curing time of the layers	10 s
Retraction speed	25 mm/min

**Table 2:** Summary of compression test results.

	Samples	Mean	S.E.
Compressive Modulus (MPa)	Honeycomb	81.62	0.60
	Re-entrant H	88.67	2.61
	Diamond	118.19	2.28
	Square	115.65	4.10
Compressive Strength (MPa)	Honeycomb	4.95	0.36
	Re-entrant H	5.68	0.34
	Diamond	6.97	0.39
	Square	6.27	0.19

## Data Availability

The data presented in this study are available on request from the corresponding author.
